# Comparing the Chinese versions of two knee-specific questionnaires (IKDC and KOOS): reliability, validity, and responsiveness

**DOI:** 10.1186/s12955-017-0814-6

**Published:** 2017-12-06

**Authors:** Chien-Chih Huang, Wen-Shiang Chen, Mei-Wun Tsai, Wendy Tzyy-Jiuan Wang

**Affiliations:** 1 Department of Physical Medicine and Rehabilitation, National Taiwan University Hospital, Taipei, Taiwan; 2 National Taiwan University, College of Medicine, Taipei, Taiwan; 3 Department of Physical Therapy and Assistive Technology, National Yang-Ming University, 155, Sec. 2, Li-Nong Street, Beitou District, Taipei, 11221 Taiwan

**Keywords:** Patient-reported outcome measurement, International knee documentation committee (IKDC), The knee injury and osteoarthritis outcome score (KOOS), Cross-cultural adaptation, Psychometric properties

## Abstract

**Background:**

The International Knee Documentation Committee Subjective Knee Form (IKDC) and the Knee Injury and Osteoarthritis Outcome Score (KOOS) are knee-specific questionnaires that have been widely used and translated into numerous languages. However, the differences in the psychometric properties between the Chinese IKDC and KOOS remain unclear. The purpose of this study was to conduct a cross-cultural adaptation of the Chinese IKDC and Chinese KOOS and to compare the psychometric properties of these two measures in patients with various knee injuries from the acute stage up to 12 weeks after receiving treatment.

**Methods:**

The original IKDC and KOOS were translated into Chinese based on the guidelines of cross-cultural adaptation and translation protocols. One hundred and seventy-three patients with various knee injuries were recruited in this study and completed both Chinese IKDC and Chinese KOOS as well as a generic health status questionnaire (Chinese Short Form-36 [SF-36]). The reliability, internal consistency, content validity, convergent and divergent validity and responsiveness of both IKDC and KOOS were assessed with appropriate indices.

**Results:**

The Chinese IKDC showed excellent reliability (ICC = 0.97) and strong internal consistency (Cronbach alpha = 0.87). The Chinese KOOS also presented good reliability with ICCs ranging from 0.89 to 0.95 and internal consistency (Cronbach alpha coefficients ranging from 0.76 to 0.97). The content validity of these two questionnaires were excellent, yielding no floor or ceiling effects. Both the Chinese IKDC and KOOS were highly associated with the physical component summary (PCS) score and weakly related to the mental component summary (MCS) score of the SF-36. Responsiveness to change was large (effect size =0.95) for the Chinese IKDC and moderate (effect sizes = 0.49~0.60) at 12-week after physical therapy.

**Conclusion:**

Both the Chinese IKDC and KOOS demonstrated good psychometric properties. However, the Chinese IKDC was more sensitive to changes over a period of 2, 4, 8, 12 weeks of physical therapy than the Chinese KOOS. The ROC analyses revealed a value of area under the curve (0.83 for the Chinese IKDC and 0.67–0.79 for the subscales of Chinese KOOS). Minimal clinically important difference values were 9.8 for the Chinese IKDC and 0.79, 0.76, 0.76, 0.76, 0.67 for the Symptoms, Pain, Activities of Daily Living, Sport/Recreation, and Quality of Life subscales of Chinese KOOS, respectively. The current study provides information for clinicians and researchers to use these appraisal tools for Chinese-speaking patients with various knee disorders.

## Background

Clinical outcome research is required to evaluate the benefits and cost effectiveness of new diagnostic, surgical, and rehabilitative approaches for treating knee problems [1]. Both performance-based and self-reported measures are often used to evaluate clinical outcomes of orthopedic patients. To be used among various language groups and in diverse countries, patient-oriented measures (represented by self-administered questionnaires) must be translated, adapted to distinct cultural characteristics, and validated using common processes to evaluate their psychometric properties [2].

In 2007, the International Knee Documentation Committee Subjective Knee Form (IKDC) and Knee Injury and Osteoarthritis Outcome Score (KOOS) were identified as the eminent instruments for assessing general knee quality of life, involving numerous questions that assess the symptoms and disabilities relevant to patients with knee disorders [3]. Quality of life measures capture the patient perspective regarding the disease and treatment, perceived need for health care, and preferences concerning treatment and outcomes [4].

The IKDC and KOOS have been translated to and validated in several languages and have been widely used to evaluate various knee injuries [5–23]. To employ these questionnaires among multiple language groups and in diverse cultural settings, they must be translated, adapted based on cultural characteristics, and validated against the original versions. The cross-cultural adaptation guidelines described by Guillemin et al. [2] are widely used to translate and adapt questionnaires. The criteria recommended for selecting instruments include the characteristics of patients for whom the instrument was developed, the instrument content, and its psychometric properties [24].

In Chinese-speaking countries, few translated knee-specific questionnaires have been validated. Questionnaires designed as subjective scoring systems can be used in various countries if they are translated and validated to target a specific language and population [2, 25]. In addition, using culturally equivalent, standardized questionnaires simplifies the problems involved in meta-analyses during clinical research, enabling the comparison of studies and minimizing the reporting bias in various countries [26, 27].

A review of the available outcome measurements for knee ligament injuries indicated that the IKDC is the preferred measurement tool [28]. The IKDC provided the optimal overall measure of the critical symptoms and disabilities of a population of postoperative articular cartilage repair patients [29]. The IKDC was found more useful than the KOOS for evaluating patients with anterior cruciate ligament (ACL) ruptures [30]; however, at present the differences in the psychometric properties between the Chinese IKDC and KOOS remain unclear. Responsiveness of an outcome measure referring to the ability to detect changes of a construct of interest over time, is considered essential. Several researchers examined the responsiveness of both the IKDC and KOOS; however, most of them provided responsiveness information for detecting changes over a period of more than 3 months. The clinical utility of these instruments on reflecting improvement of patient’s status in a shorter period of time (<3 months) is unknown. Therefore, the purposes of this study were to (1) translate the English versions of the IKDC and KOOS into Chinese versions based on cross-cultural adaptation guidelines, (2) to evaluate and compare the reliability and validity of the Chinese IKDC and Chinese KOOS in patients with a variety of knee conditions, and (3) to determine and compare the responsiveness of the Chinese IKDC and KOOS in detecting clinical changes over 4 time points (2-, 4-, 8-, and 12-week) following treatment.

## Methods

### Cross-cultural adaptation process

The IKDC and KOOS were translated and adapted from English to traditional Chinese versions by using a forward-backward translation protocol based on the guidelines of Guillemin et al. and the recommendations for the cross-cultural adaptation of health status measures of the American Academy of Orthopaedic Surgeons (AAOS) [2].

Two independent bilinguals (native Chinese speakers) translated the original English versions of IKDC and KOOS into Chinese. The informed translator was a physical therapist who possessed 20 years of clinical experience treating adults with orthopedic disorders, and the uninformed translator was a computer engineer. A consensus meeting was held to resolve the discrepancies between the two translations of both questionnaires. The synthesized versions for both the IKDC and KOOS were produced after making several word expression amendments. Then, the synthesized Chinese versions of two questionnaires were back-translated into English by two independent bilinguals (native English speakers) who were blind to the original versions of both questionnaires. None of the back translators was aware or informed of the concepts used in the questionnaires.

An expert committee comprising 2 forward translators, 2 backward translators, a methodologist, a clinician (rehabilitation physician) and a language specialist reviewed all the translation versions of both questionnaires to assure the semantic, idiomatic, experiential, and conceptual equivalence between languages. After consolidating all the versions of the questionnaires, the committee developed the pre-final versions of Chinese IKDC and Chinese KOOS for pre-testing. Ten patients with various knee pathologies participated in the pre-testing step of the validation process and completed the pre-final versions of two questionnaires. Comprehensibility and cultural relevance of the questionnaire items were discussed with the subjects through a face-to-face interview.

### Participants

The Research Ethics Committee at National Taiwan University Hospital approved this clinical trial. A convenience sample of 173 patients with various knee injuries undergoing physical therapy at the Department of Physical Medicine and Rehabilitation, National Taiwan University Hospital were recruited for participating in this study (Table 1). All patients provided their informed consents before entering in the study. Patients were excluded if they exhibited other joint problems affecting the lower extremities or back, systematic inflammatory rheumatic disease, neurological or vascular conditions, or psychiatric disorders. During the study period, all patients received physical therapy including physical agents, joint mobilization, stretching and strengthening exercises, etc. for their knee symptoms.

**Table 1:** Characteristics of the Subjects

Variables	*N* (%)		Mean (SD)
Age (years)			44.4 (19.0)
Gender			
Female	94 (54.3)		
Male	79 (45.7)		
Height (cm)			164.3 (8.4)
Weight (kg)			63.4 (10.1)
BMI (kg/m^2^)			23.5 (3.1)
Side of the affected knee			
Right	67 (38.7)		
Left	75 (43.4)		
Bilateral	31 (17.9)		
Diagnosis		Female (%)	Duration since onset (month)
Osteoarthritis	63 (36.4)	46 (73.0)	13.2
Ligament injury			
Anterior cruciate ligament (ACL)	39 (22.5)	10 (25.6)	8.6
Posterior cruciate ligament (PCL)	14 (8.1)	7 (50.0)	19.5
Medial collateral ligament	2 (1.2)	1 (50.0)	9.5
Patellofemoral injury	14 (8.1)	4 (28.6)	14.9
Patella fracture	4 (2.3)	3 (75.0)	5.3
Meniscal/Cartilage injury	10 (5.8)	4 (40.0)	9.0
Knee sprain	19 (11.0)	12 (62.3)	4.2
Knee contracture	4 (2.2)	3 (75.0)	8.3
Post total knee arthroplasty	4 (2.2)	4 (100.0)	4.3

### Instruments

Two disease-specific questionnaires, the Chinese IKDC and Chinese KOOS, the Chinese SF-36 [31] and a 15-point global rating of change (GROC) scale [32] were used in this study. The IKDC was originally designed to measure the symptoms and functional limitations in sports activities caused by various knee impairments [33]. High IKDC scores indicate a low level of symptoms and a high level of function, whereas low scores indicate a high level of symptoms and a low level of function. Thus, a score of 100 indicates no symptoms and no limitations regarding daily or sports activities [33].

The original KOOS questionnaire is an extension of the Western Ontario and McMaster Universities Arthritis Index, and is a well-designed, and simple self-administered instrument that was developed to assess the short- and long-term symptoms and function of patients with knee injuries and osteoarthritis [34]. It is a 42-item disease-specific questionnaire comprising 5 subscales: symptoms, pain, activities of daily living (ADL), sports and recreation (Sport/Rec) and knee-related quality of life (QOL). The raw scores are separately calculated for each subscale and transformed to a 0–100 scale on which 0 indicates severe problems and 100 indicates no problems [20]. The KOOS questionnaire has demonstrated reliability, validity, and responsiveness among distinct populations exhibiting varying pathologies, injury durations, ages, and activity levels [5, 6, 8, 9, 19–21, 23, 34].

The SF-36 comprises 8 subscales: physical functioning (PF), role-physical (RP), bodily pain (BP), general health (GH), vitality (VT), social functioning (SF), role-emotional (RE), and mental health (MH) [35]. The PF, RP, and BP scales are most highly correlated with the physical component summary (PCS), contributing the most to the PCS score. The MH, RE, and SF scales are most highly correlated with the mental component summary (MCS), contributing the most to the MCS score. The VT, GH, and SF scales are notably correlated with both the PCS and MCS [35]. These 8 subscales are scored from 0 to 100, where high scores indicate a superior health status [36, 37]. The Chinese version of the SF-36 has been validated for use in Taiwan [31, 38].

A 15-point (−7~ + 7) GROC was used to monitor changes occurred between two time points [32]. The scale ranges from −7 (a very great deal worse) through 0 (no change) to +7 (a very great deal better) with the score of +4 or more representing moderately better (+4), a good deal better (+5), a great deal better (+6), or a very great deal better (+7). For the test-retest reliability analysis, patients scoring between 2 (a little bit better) and −2 (a little bit worse) were considered to have stable clinical states and included for analysis. GROC has also been used to measure the subject’s impression of the change following an intervention. A cutpoint can be chosen to dichotomize patients as achieving significant improvement or not.

### Procedure

The Chinese IKDC, Chinese KOOS, and Chinese SF-36 were administered to the study participants during their first visits to the outpatient department. Before the first treatment, 173 patients completed the Chinese IKDC, Chinese KOOS, and the Chinese SF-36 questionnaires. To assess the test-retest reliability, 40 patients (mean age, 43 y) filled out the Chinese IKDC and Chinese KOOS again after a 5-to-7-day interval. The GROC was also rated by this patient cohort. This interval was long enough for the patients to have forgotten previous responses but not so long that their condition would have changed. To minimize the clinical changes, no treatment was provided to these patients over the test-retest interval.

For the purpose of analyzing the responsiveness of the Chinese IKDC and KOOS, follow-up reassessments were performed at 2, 4, 8, and 12 weeks following treatment. In addition, the GROC was administered again at the 12-week follow-up as the external criterion for indicating significant improvement with treatment.

### Data management and analysis

The statistical analysis was conducted using SPSS version 20.0 (SPSS Inc., Chicago, IL). The Kolmogorov–Smirnov test was used for normality check of all scores. The level of significance for all statistical procedures was *P* < 0.05.

The interpretability was evaluated by assessing the occurrence and distribution of floor and ceiling effects regarding baseline scores. A floor or ceiling effect of <15% was considered to be acceptable, which means that less than 15% of the respondents achieve the minimum or maximum possible scores [39]. Skewness statistics are usually evaluated informally; values < −1 or > +1 signal substantially non-normal distributions potentially in need of additional evaluation [40].

### Reliability analysis

The test-retest reliability was assessed by the intraclass correlation coefficient (ICC), using a two-way mixed effects model for absolute agreement [41]. ICCs of 0.70 or greater indicated acceptable test-retest reliability (0.81~0.90, good; 0.91~1.0, excellent) [7, 42].

To determine the measurement precision, the standard error of measurement (SEM) was calculated by multiplying the square root of 1 minus the ICC by the standard deviation (SD) of the baseline score of the instrument. The minimum detectable change (MDC) based on the 95% confidence interval of SEM was then computed with multiplying the SEM by 1.96 and the square root of 2 [43].

The internal consistency of the first administration of each questionnaire was calculated using the Cronbach alpha to estimate the average correlations among items within a subscale [44]. An alpha value of 0.70 or greater indicated satisfactory internal consistency [45]; however, a value greater than 0.95 could indicate redundancy of one or more items [39].

### Validity analysis

Construct validity refers to the degree to which the questionnaire measures the characteristic to be measured. We tested the construct validity of the Chinese IKDC and KOOS by calculating the Spearman’s correlation coefficients of the two instruments with the Chinese SF-36 scores. Convergent and divergent validity were both assessed [46]. It was hypothesized a priori that the correlations between the IKDC and KOOS subscales with the SF-36 subscales of physical health (PF, RP, BP, and PCS) should be strong (convergent validity) while the correlations of the IKDC and KOOS subscales with the SF-36 subscales of mental health (GH, VT, SF, RE, MH, and MCS) should be weak (divergent validity). Spearman’s correlation coefficients of >0.50, 0.35~0.50, and <0.35 were considered strong, moderate, and weak, respectively [23].

### Responsiveness analysis

Responsiveness of two instruments was assessed by the effect size (ES) as well as the receiver operating characteristic (ROC) curve method. ES is calculated by the difference between the mean baseline and follow-up scores of a measure, divided by the standard deviation (SD) of its baseline score [47]. Four ESs were computed for the Chinese IKDC and 5 subscales of Chinese KOOS at 4 time points of follow-up. An ES value between 0.20 and 0.50 represents a change of approximately one-fifth of the baseline SD and is considered small; between 0.51 and 0.80 reflects a change of at least half the baseline SD and is considered moderate; an ES value of 0.80 or greater represents a change of at least four-fifths the baseline SD and is considered large [47]. Larger ES indicates a greater ability to detect clinical changes. Pair-t tests were used to compare the baseline and follow-up scores of Chinese IKDC and KOOS following 2, 4, 8, and 12 weeks of physical therapy treatment.

The ROC curve analysis was used to establish the minimal clinically important difference (MCID) scores for Chinese IKDC and the subscales of Chinese KOOS. The score of GROC ≥ 4 (moderately better) was chosen as the cutoff point for discriminating between patients who perceived themselves to achieve significant improvement from those who did not. The optimal cut off point was computed using the Youden index and taken as the MCID, which indicated the change score associated with the least misclassification [48]. For each value of change of the Chinese IKDC and the subscales of Chinese KOOS, the sensitivity and specificity were calculated and used to plot the ROC curves: the sensitivity values and false-positive rates (1-specificity) were plotted on the y and the x axis of the curve, and the area under the curve (AUC) showed the probability that a measure correctly classifies patients as either meaningfully improved or not. An AUC of more than 0.70 is considered to be acceptable.

## Results

### Cross-cultural adaptation process

During the translation and adaptation stages, the pre-final versions of both the Chinese IKDC and KOOS were well accepted by subjects in the pre-testing. All subjects completed both questionnaires without missing items and demonstrated a clear understanding of the scale items. No major conceptual or cultural differences were found between the Chinese and English-speaking populations. Therefore, the pre-final versions of Chinese IKDC and KOOS were not modified further and were considered the final versions. To complete the final step of cross-cultural adaptation, the Chinese IKDC was submitted to the developer. It is now available for download at the website of the American Orthopaedic Society for Sports Medicine (AOSSM): https://www.sportsmed.org/AOSSMIMIS/members/downloads/research/IKDCChineseTraditional.pdf [49].

### Interpretability

The mean, standard deviation (SD), median, mode, minimum, maximum, and skewness for the Chinese IKDC and 5 KOOS subscales have been shown in Table 2. The Chinese IKDC scores indicated a normal distribution and negligible numbers of patients who demonstrated floor or ceiling effects. The Chinese KOOS scores were also distributed normally. In addition, the percentage of subjects who received the minimum possible scores in the subscales Sport/Rec and QOL were 8.7 and 1.7%, respectively. Maximum possible scores in the subscales symptoms, pain, ADL, Sport/Rec and QOL were 0.6, 0.6, 4.6, 1.7 and 0.6%, respectively. We consider that no ceiling or floor effect occurred in the Chinese KOOS.

**Table 2:** Baseline Scores of 173 Subjects for the Chinese IKDC and the subscales of the Chinese KOOS

	Mean (SD)	Median	Mode	Minimum	Maximum	Skewness
IKDC^a^	51.6 (16.0)	52.9	54.0	13.8	97.7	0.02
KOOS^b^						
Symptoms	68.3 (17.9)	72.2	75.0	8.3	100.0	−0.69
Pain	63.3 (18.6)	64.3	57.1	21.4	100.0	−0.37
ADL	74.1 (19.6)	79.4	91.1	5.9	100.0	−1.10
Sport/Rec	42.4 (26.3)	40.0	60.0	0.0	100.0	0.07
QOL	43.0 (18.7)	43.8	43.8	0.0	100.0	0.04

^a^ IKDC: International Knee Documentation Committee Subjective Knee Form

^b^ KOOS: Knee Injury and Osteoarthritis Outcome Score

### Reliability analysis

Over the 5-to-7 day interval, 32 out of 40 subjects were considered to have remained stable (−2 to +2 on the GROC). Eight patients reported that their scores were ≥3, and excluded in the test-retest reliability analysis. Of the 32 patients, 8 (24.2%) exhibited osteoarthritis, 8 (24.2%) ACL injuries, 4 (12.1%) patellofemoral pain syndrome, 3 (9.1%) posterior cruciate ligament (PCL) injuries, 3 (9.1%) nonspecified knee sprain, and one patient each exhibited derangement (3.0%), medial collateral ligament injury (3.0%), patellar fracture (3.0%), meniscal and cartilage injuries (3.0%), tendinitis and bursitis (3.0%), total knee arthroplasty (3.0%), and PCL reconstruction (3.0%).

The test-retest reliability was excellent for the Chinese IKDC with an ICC of 0.97 (*P* < 0.001). Good test-retest reliabilities were also found in the Chinese KOOS questionnaire with the ICCs of 0.89 or higher (Table 3).

**Table 3:** Test-retest Reliability and Validity of the Chinese IKDC score and the Subscales of Chinese KOOS

	Mean 1 (SD)	Mean 2 (SD)	ICC^c^	SEM^d^	MDC^e^	Cronbach Alpha^f^
IKDC^a^	56.9 (17.7)	58.2 (17.1)	0.97	3.2	8.9	0.87
KOOS^b^						
Symptoms	70.9 (20.9)	72.1 (20.5)	0.89	7.0	19.4	0.76
Pain	65.1 (19.8)	64.7 (19.9)	0.91	6.0	16.6	0.88
ADL	77.1 (22.4)	78.1 (20.1)	0.93	6.2	17.0	0.97
Sport/Rec	49.5 (29.2)	51.4 (28.1)	0.91	8.8	24.3	0.91
QOL	43.8 (22.4)	45.1 (22.6)	0.95	5.1	14.2	0.77

^a^IKDC: International Knee Documentation Committee Subjective Knee Form

^b^KOOS: Knee Injury and Osteoarthritis Outcome Score

^c^ICC: Intraclass Correlation Coefficient

^d^SEM: Standard Error of Measurement

^e^MDC: Minimum Detectable Change

^f^Based on first measurement (*n* = 173)

The SEM and MDC values of the Chinese IKDC were 3.2 and 8.9, which was smaller than the SEM and MDC of the 5 subscales Chinese KOOS (SEM range: 5.1~8.8; MDC range: 14.2~24.3) (Table 3).

The Chinese IKDC demonstrated a high internal consistency, yielding a Cronbach alpha value of 0.87. Moderate to high internal consistency was also found in the Chinese KOOS subscales with the values ranging from 0.76 to 0.97 (Table 3).

### Validity analysis

Table 4 shows the correlations among the Chinese IKDC, 5 subscales of Chinese KOOS, and the Chinese SF-36 scores.

**Table 4:** Spearman Correlations Between the Chinese IKDC and the Subscales of Chinese KOOS

	IKDC^a^	KOOS^b^				
		Symptoms	Pain	ADL	Sport/Rec	QOL
SF-36						
Physical functioning	0.79^d^	0.60^d^	0.57^d^	0.70^d^	0.67^d^	0.54^d^
Role physical	0.54^d^	0.42^d^	0.48^d^	0.48^d^	0.43^d^	0.41^d^
Bodily pain	0.63^d^	0.53^d^	0.41^d^	0.56^d^	0.45^d^	0.45^d^
General health	0.35^d^	0.30^d^	0.20^d^	0.34^d^	0.23^d^	0.35^d^
Vitality	0.25^d^	0.18^c^	0.13	0.19^c^	0.18^c^	0.30^d^
Social functioning	0.45^d^	0.38^d^	0.40^d^	0.43^d^	0.40^d^	0.47^d^
Role emotional	0.34^d^	0.28^d^	0.29^d^	0.35^d^	0.17^c^	0.30^d^
Mental health	0.29^d^	0.23^d^	0.22^d^	0.25^d^	0.19^c^	0.36^d^
Physical composite score	0.74^d^	0.59^d^	0.55^d^	0.68^d^	0.57^d^	0.53^d^
Mental composite score	0.21^d^	0.17^c^	0.17^c^	0.20^d^	0.09	0.29^d^
IKDC	1.00	0.70^d^	0.63^d^	0.79^d^	0.78^d^	0.58^d^

^a^IKDC: International Knee Documentation Committee Subjective Knee Form

^b^KOOS: Knee Injury and Osteoarthritis Outcome Score

^c^Correlation is significant at the 0.05 level (2-tailed)

^d^Correlation is significant at the 0.01 level (2-tailed)

### Responsiveness analysis

Table 5 shows the mean baseline scores, mean scores after treatment, and ES for the Chinese IKDC and KOOS at the 2-, 4-, 8-, and 12-week follow-up. The Chinese IKDC demonstrated relatively larger responsiveness than did the 5 subscales of Chinese KOOS at 4 time points of follow-up.

**Table 5:** Baseline Mean Scores (SD), Mean Scores after Treatment, Effect Sizes (ES) of the Chinese IKDC and the subscales of Chinese KOOS at 2, 4, 8, 12 Weeks

	IKDC^a^	KOOS^b^				
		Symptoms	Pain	ADL	Sport/Rec	QOL
Baseline						
Mean	46.4	65.0	61.1	69.9	37.2	40.9
SD	15.4	16.8	18.42	19.3	26.4	14.8
Week 2						
Mean	52.2	67.1	63.8	71.7	40.2	42.0
*P* value	<0.001^c^	0.244	0.147	0.323	0.256	0.499
ES	0.37	0.12	0.15	0.09	0.12	0.07
Week 4						
Mean	53.5	68.6	64.8	73.3	44.7	44.3
P value	<0.001^c^	0.131	0.124	0.156	0.015^c^	0.081
ES	0.46	0.21	0.23	0.18	0.28	0.23
Week 8						
Mean	55.8	71.0	67.4	76.2	47.5	46.1
P value	<0.001^c^	0.021^c^	0.018^c^	0.017^c^	0.005^c^	0.048^c^
ES	0.61	0.35	0.37	0.33	0.39	0.35
Week 12						
Mean	61.1	73.9	70.5	79.6	52.9	48.2
P value	<0.001^c^	0.004^c^	0.003^c^	0.001^c^	<0.001^c^	0.010^c^
ES	0.95	0.53	0.54	0.50	0.60	0.49

^a^IKDC, International Knee Documentation Committee Subjective Knee Form

^b^KOOS, Knee Injury and Osteoarthritis Outcome Score

^c^Paired-t test: significant at the 0.05 level (2-tailed)

The ESs of the Chinese IKDC were moderate (0.61) after the 8-week follow-up and large (0.95) at the 12-week follow-up while the Chinese KOOS subscales only demonstrated moderate ESs (0.49–0.60) at the 12-week follow-up. At the 2-, 4-, 8-week follow-up, only the Chinese IKDC score had moderate effect size (0.61 at 8-week follow-up), the rest scores of Chinese IKDC and all of the Chinese KOOS demonstrated small effect sizes (0.09~0.46).

Results of the within-group comparisons showed that at 8- and 12-week follow-up, the differences of both the Chinese IKDC and all of the Chinese KOOS subscales were statistically significant. At the 2- and 4-week time points, even with small effect sizes (0.37 and 0.46), the differences of the Chinese IKDC scores from the baseline were still significant, while the differences of the Chinese KOOS subscales were mostly nonsignificant.

The area under the ROC curve, minimum clinically important difference, and the sensitivity and specificity for the minimum clinically important differences are displayed in Table 6. At 12 weeks after intervention, the area under the ROC curve was significantly different from 0 for the Chinese IKDC and all of the Chinese KOOS subscales. The AUC of Chinese IKDC (0.83) was larger than all of the KOOS subscales (0.67~0.79). The AUCs of Chinese KOOS subscales were good except for the QOL subscale (0.67) which was less than optimal.

**Table 6:** Results of the Receiver Operating Characteristic Curve Analysis

	Area Under Curve (95% Confidence Interval)	MCID	Sensitivity	Specificity
IKDC	0.83* (0.76–0.89)	9.8	0.79	0.72
KOOS				
Symptoms	0.79* (0.72–0.87)	10.9	0.67	0.76
Pain	0.76* (0.68–0.84)	16.1	0.51	0.89
ADL	0.79* (0.72–0.86)	8.1	0.70	0.76
Sport/Rec	0.79* (0.72–0.86)	12.5	0.77	0.67
QOL	0.67* (0.58–0.77)	9.4	0.56	0.75

*MCID* minimal clinically important difference, *IKDC* International Knee Documentation Committee Subjective Knee Form, *KOOS* Knee Injury and Osteoarthritis Outcome Score

*P ≤ 0.001

## Discussion

In this study, the original versions of the IKDC and KOOS were translated and validated to facilitate assessing Chinese-speaking patients with a variety of knee injuries. To our knowledge, our study is the first one to concurrently examine and compare the psychometric properties of the Chinese IKDC and KOOS. When assessing various knee injuries, both the Chinese IKDC and Chinese KOOS demonstrated excellent reliability and good validity. Consistent with the findings of other studies, Chinese IKDC and Chinese KOOS are reliable and valid instruments. Cross-culture adapted instruments are valuable, especially when international comparisons need to be made.

The SEM and MDC analysis indicated that the Chinese IKDC values (SEM 3.2; MDC 8.9) were smaller compared with the American data from Greco et al. [43] at 6 (SEM 5.6; MDC 15.6) and 12 (SEM 4.9; MDC 13.7) months after surgery, Dutch version [50] (SEM 5.3; SDD 14.6), and Turkish version [7] (SEM 6.0; SDD 16.4); comparable with Irrgang et al. [33] (SEM 4.6; MDC 9.0); but larger than the American data from Crawford et al. [51] (SEM 3.2; MDC 8.8) and Brazilian version [14] (SEM 2.4; MDC 6.7). The Chinese KOOS values (SEM 5.1~10.6; MDC 10.4~29.4) were smaller compared with those of the Dutch version [50] (SEM 7.0~12.6; SDD 19.4~35.0); but larger than the Persian version [52] (SEM 2.1~3.1; MDC 5.8~8.5) and the Polish version [19] (SEM 3.9~7.3; MDC 10.9~20.2). In summary, our data are in agreement with results from other versions from different countries and support the responsiveness of both the Chinese IKDC and KOOS questionnaires as outcome measures for various knee conditions.

The internal consistency of the Chinese IKDC was high (Cronbach alpha, 0.87), demonstrating similarity to that of the original version of the IKDC (Cronbach alpha, 0.92) [33]. Satisfactory Cronbach alpha values were also found in the Chinese KOOS symptoms subscale (0.76), QOL subscale (0.77), pain subscale (0.88), and Sport/Rec subscale (0.91). Our data is similar to the Swedish version of KOOS [53], in which the Cronbach alpha values were 0.74 for the symptoms subscale and 0.71 for the QOL subscales. Results of this study also showed that the Chinese KOOS ADL subscale had the highest Cronbach alpha (0.97), similar to the value (0.95) of the Swedish KOOS ADL subscale [53]. However, since these Cronbach alpha values were greater than 0.95, redundancy of items may exist in Chinese KOOS ADL subscale.

Following the same validation process of the original IKDC, we used the Chinese SF-36 to evaluate the construct validity of both the Chinese IKDC and KOOS questionnaires. Strong correlations (Spearman’s rhos = 0.54~0.79) between the Chinese IKDC and the physical health dimensions of the Chinese SF-36 confirms the convergent validity of the Chinese IKDC. Weak correlations (Spearman’s rhos = 0.21~0.34) between the Chinese IKDC and the mental function dimensions of the SF-36 supports the divergent validity of the Chinese IKDC. The strong correlations between the Chinese IKDC and the PF and BP domains of the Chinese SF-36 demonstrated values comparable to the original IKDC and other translated questionnaires [7, 10, 12, 13, 17, 33].

Our results showed that the correlations between the role physical subscale of the Chinese SF-36 and each of the 5 subscales of the Chinese KOOS were all moderate (Spearman’s rhos = 0.41~0.48). The calculated correlation coefficients of 0.70 (between the Chinese KOOS ADL and Chinese SF-36 PF), 0.43 (between the Chinese KOOS Sport/Rec and Chinese SF-36 RP), and 0.45 (between the Chinese KOOS Sport/Rec and Chinese SF-36 BP) were similar to the values 0.68, 0.43, and 0.43, respectively, calculated in a study on the Swedish version of the KOOS [53].

Both instruments demonstrated their largest changes occurred at the end test time point (12-week follow-up), although only the Chinese IKDC had large effect size (0.95), while most of the KOOS subscales demonstrated moderate effect sizes (0.49~0.60). The Chinese IKDC demonstrated larger effect sizes compared with each of the Chinese KOOS subscales at the 2-, 4-, 8-, and 12-week follow-up; therefore, we believe that the Chinese IKDC was more responsive to changes over time than the Chinese KOOS.

The ES of the Chinese IKDC at week 12 was similar to that in the study of Irrgang et al. [54] (ES = 1.13; 207 patients with various knee problems at 19-month follow-up) and Greco et al. [43] (ES = 1.06; 72 patients with focal articular cartilage defects at 12-month follow-up). Interpretation of the responsiveness indices point to the Chinese IKDC as the instrument of choice for evaluating clinical changes occurred less than 12 weeks.

The responsiveness analysis showed that both the Chinese IKDC and KOOS were able to detect change over time. Increased effect sizes over time observed in the Chinese IKDC and KOOS subscales were expected because all of the participants were under physical therapy treatment.

As far as we know, there is no other study that has examined the responsiveness of the IKDC and the KOOS at 2, 4, and 8-week after conservative treatments. These time intervals for responsiveness were chosen because they corresponded to common treatment durations of physical therapy for knee disorders. Improvement from the commencement of treatment was expected along a time line. It should be detected by a reliable, valid, and responsive outcome measure. Therefore, the responsiveness information regarding shorter (less than 12 weeks) time intervals still have a certain degree of reference for clinical application and research.

In this study, we successfully constructed the data on the AUC and MCID of the Chinese IKDC and KOOS. The MCID score (9.8) of the Chinese IKDC during the 12 weeks follow-up was smaller compared with those of Irrgang et al. [54] (MCID = 11.5 and 20.5; 207 patients with various knee injuries at the 19-month follow up), but larger than that of Greco et al. [43] (MCID = 6.3; 72 patients with focal articular cartilage defects at the 6-month follow up). The Chinese KOOS yielded an AUC (0.67~0.79) and MCID (8.1~16.1) at the 12 weeks follow-up. Our findings of the AUC for each of the Chinese KOOS subscales (0.79, 0.76, 0.76, 0.76, 0.67) was smaller than the Italian KOOS subscales (0.88, 0.89, 0.94, 0.93, and 0.85) for the Symptoms, Pain, Activities of Daily Living, Sport/Recreation, and Quality of Life subscales, respectively [55]. The MCIDs of the Chinese KOOS subscales on Symptoms, Pain, and Sport/Recreation (10.9, 16.1, 12.5) were comparable to the Italian KOOS (10.7, 16.7, 12.5; 148 patients undergoing a 4-week rehabilitation program after total knee arthroplasty), while the MCIDs of the Chinese KOOS subscales on Activities of Daily Living and Quality of Life were smaller than those in the Italian KOOS (18.4, 15.6) [55]. Since the MCIDs may vary with patient groups, clinical characteristics, and analytical approaches, interpretations of the study findings need to be cautious.

Comparisons between the Chinese IKDC and KOOS in their psychometric properties have been made in several studies [30, 50]. A cross-sectional cohort study involving patients who were on the waiting list for meniscal surgery, and patients between 6 weeks and 6 months after meniscal surgery showed favorable results for reliability and validity of the Dutch IKDC compared with the Dutch KOOS. Despite a tendency toward the KOOS as the outcome measure for meniscal injuries, the author suggests that the IKDC Subjective Knee Form is the best applicable instrument for patients with meniscal injuries [50]. van Meer et al. conducted another study to compare the Dutch IKDC and the Dutch KOOS in a group of patients with recent anterior cruciate ligament ruptures. Their results showed that all KOOS subscales and the IKDC had good reliability. However, the KOOS did not perform optimally on the following measurement properties: relevance of the questions, construct validity, responsiveness, and ceiling effects, while the IKDC satisfied the criteria for all properties in this specific group of patients. They concluded that the Dutch IKDC is more useful than the Dutch KOOS questionnaire to evaluate patients with ACL injuries [30].

This study has some limitations. First, considering the heterogeneity in diagnosis of the study participants, comparison of the psychometric properties among various knee-injury groups for the Chinese IKDC and KOOS could not be made. However, these two instruments are considered the site-specific patient-reported outcome measures and are intended to measure the same construct for patients with a variety of knee problems. When testing the same group of patients with these two questionnaires at the same time, we could still compare the measurement properties between them. Further studies evaluating different subgroups of knee injury will be needed before generalizations are applicable. Second, the responsiveness follow-up period of 12 weeks was relatively short; this measure should not be used to infer the 6-month (medium term) and 12-month (long term) outcomes. We recommend conducting further studies to compare the Chinese IKDC and KOOS by using various patient groups and extended follow-up times.

## Conclusion

The Chinese IKDC and KOOS were both culturally adapted and validated in a group of Chinese-speaking patients with various knee injuries. Both the Chinese IKDC and KOOS demonstrated high levels of reliability and validity. However, the Chinese IKDC showed better performance on the psychometric properties including ICC, SEM, MDC, and Cronbach alpha than the Chinese KOOS. Chinese IKDC was also more sensitive to changes over a period of 2, 4, 8, 12 weeks of treatment than the Chinese KOOS. Both Chinese IKDC and most of the Chinese KOOS subscales demonstrated good discriminative capacities in detecting clinically meaning changes occurred at 12 weeks after treatment. The MCIDs of the two instruments were also revealed in this study. The current study provides information for clinicians and researchers to use these appraisal tools for Chinese-speaking patients with various knee disorders.

## Abbreviations

AAOS: The American Academy of Orthopaedic Surgeons
ACL: Anterior cruciate ligament
AUC: Area under curve
BP: Bodily pain
ES: Effect size
GH: General health
ICC: Intraclass correlation coefficient
IKDC: International knee documentation committee subjective knee form
KOOS: The knee injury and osteoarthritis outcome score
MCID: The minimal clinically important difference
MCS: The mental component summary
MDC: The minimum detectable change
MH: Mental health
PCL: Posterior cruciate ligament
PCS: Physical component summary score
PF: Physical functioning
QOL: Quality of life
RE: Role-emotional
RP: Role-physical
SD: Standard deviation
SEM: Standard error of measurement
SF: Social functioning
SF-36: The 36-item short form health survey
VT: Vitality
